# Weight Loss Programs May Have Beneficial or Adverse Effects on Fat Mass and Insulin Sensitivity in Overweight and Obese Black Women

**DOI:** 10.1007/s40615-014-0006-6

**Published:** 2014-03-05

**Authors:** Benjamin Leon, Bernard V. Miller, Gloria Zalos, Amber B. Courville, Anne E. Sumner, Tiffany M. Powell-Wiley, Mary F. Walter, Myron A. Waclawiw, Richard O. Cannon

**Affiliations:** 1 Cardiovascular and Pulmonary Branch, National Heart, Lung, and Blood Institute, Building 10-CRC Room 5-3330, 10 Center Drive, Bethesda, MD 20892 USA; 2 Office of Biostatistics Research, National Heart, Lung, and Blood Institute, Bethesda, MD USA; 3 Diabetes, Endocrinology and Obesity Branch, Clinical Center; National Institutes of Health, Bethesda, MD USA; 4 Core for Clinical Laboratory Services, Clinical Center; National Institutes of Health, Bethesda, MD USA; 5 Nutrition Department, Clinical Center; National Institutes of Health, Bethesda, MD USA

**Keywords:** Obesity, insulin sensitivity, women, race, intervention

## Abstract

**Objective:**

Weight loss interventions have produced little change in insulin sensitivity in black women, but mean data may obscure metabolic benefit to some and adverse effects for others. Accordingly, we analyzed insulin sensitivity relative to fat mass change following a weight loss program.

**Design and Methods:**

Fifty-four black women (BMI range 25.9 to 54.7 kg/m^2^) completed the 6-month program that included nutrition information and worksite exercise facilities. Fat mass was measured by dual-energy X-ray absorptiometry, and insulin sensitivity index (S_I_) was calculated from an insulin-modified intravenous glucose tolerance test using the minimal model.

**Results:**

Baseline S_I_ (range 0.74 to 7.58 l/mU^−1^•min^−1^) was inversely associated with fat mass (*r* = −0.516, *p* < 0.001), independent of age. On average, subjects lost fat mass (baseline 40.8 ± 12.4 to 39.4 ± 12.6 kg [mean ± SD], *P* < 0.01), but 17 women (32 %) actually gained fat mass. S_I_ for the group was unchanged (baseline 3.3 ± 1.7 to 3.2 ± 1.6, *P* = 0.67). However, the tertile with greatest fat mass loss (−3.6 kg, range −10.7 to −1.7 kg) improved insulin sensitivity (S_I_ +0.3 ± 1.2), whereas the tertile with net fat mass gain (+0.9 kg, range −0.1 to +3.8 kg) had reduced insulin sensitivity (S_I_ −0.7 ± 1.3) from baseline values (*P* < 0.05 by ANOVA).

**Conclusions:**

Black women in a weight loss program who lose fat mass may have improved insulin sensitivity, but fat mass gain with diminished sensitivity is common. Additional support for participants who fail to achieve fat mass loss early in an intervention may be required for success.

## Introduction

The obesity epidemic has steadily worsened as nearly 70 % of adults in the USA are at least overweight and more than one-third are obese [1, 2]. The prevalence of obesity is the highest among underrepresented minority populations, particularly blacks who have the highest age-adjusted rate of obesity nationwide [1, 2]. Additionally, blacks also have a disproportionately high prevalence of type 2 diabetes and are at greater risk of developing associated cardiovascular complications including coronary artery disease, myocardial infarction, stroke, congestive heart failure, and peripheral vascular disease [3–6].

Major contributors to the growing obesity epidemic are inactivity and excess energy intake [7]. Evidence suggests that the work place contributes to the prevalence of obesity as employees in the USA are sedentary for large portions of the workday [7, 8]. To counter the mounting obesity rates, many organizations have initiated wellness programs at the work site to achieve weight loss by encouraging exercise and reduced caloric intake [9–14]. While many of these programs have been effective in promoting weight loss and improving health measures among whites, black women have been less successful in achieving these ends [15]. Therefore, we hypothesized that an intervention at the worksite that provides women with healthful information, either through interactive group sessions or internet-based tools, as well as exercise resources in the work place would enable fat mass loss and improve insulin-mediated glucose metabolism in overweight and obese black women. Specifically, we proposed that this decrease in fat mass would improve insulin sensitivity for those women who completed the diet and exercise program.

## Methods and Procedures

### Study population

Overweight (body mass index [BMI] 25 to <30 kg/m^2^) and obese (BMI ≥ 30 kg/m^2^) nondiabetic (fasting glucose <126 mg/dL) black (by self-report) female employees of the National Institutes of Health (NIH), Bethesda, Maryland were enrolled. Participants were recruited by flyers distributed across the Bethesda campus of NIH and self-identified as healthy without participation in structured exercise or weight loss programs and weight stable (fluctuation in weight < 5 %) over the previous 3 months. Women were excluded from participation if screening blood work revealed anemia (hemoglobin <11 g/dL), liver, kidney, or thyroid disease. Prescription medications at stable doses for at least 2 months – including hormonal preparations for thyroid dysfunction or estrogen preparations (i.e., birth control or postmenopausal hormone therapy) – were permitted, but a change in medications during the study was prespecified as an exclusion criterion from further participation due to the potential of confounding main outcome measures. The protocol was approved by the Institutional Review Board of the National Heart Lung and Blood (NHLBI) and registered in www.ClinicalTrials.gov (NCT00666172) prior to study initiation. All subjects provided informed consent.

### Study Design

All participants were provided internet-based nutrition and exercise information created by NHLBI for employees (recent version can be found at http://apps.nhlbi.nih.gov/keepthebeat) that included recommendations from the Department of Health and Human Services and the US Department of Agriculture [16]. The web site included walking paths around the NIH Bethesda campus, sample menus, healthful lifestyle information, and tools for counting calories. Each participant was given a pedometer (Walk4Life, Plainfield, IL) with instructions to increase average daily step count by 5,000 steps over their baseline average and given card-key access to private fitness rooms located in three buildings on campus, each equipped with aerobic exercise equipment (e.g., treadmill, elliptical machine, and supine bicycle). Participants were also encouraged to continue physical activity – especially walking – on nonwork days. Enrollees were randomized into either intervention or control groups: Intervention participants attended nutrition education sessions (weekly for the first 3 months, monthly for the last 3 months) conducted by a registered dietitian, whereas control subjects were not provided this instruction. Subjects were compensated for their time and inconvenience of testing.

### Testing

All tests were performed at the NIH Clinical Center, Bethesda, Maryland at baseline and at 6 months following an overnight fast. Premenopausal women were scheduled for testing within the first 10 days of their menstrual cycle. Baseline and 6 month visits included collection and review of 3-day food records by the registered dietitians. The food records, which included two work days and one nonwork day, were reviewed for accuracy by dietitians and then analyzed using the Nutrition Data System for Research (NDS-R versions 2009–2011, Minneapolis, MN) [17].

Body weight (to the nearest 0.1 kg, Scale-Tronix 5702, Carol Stream, IL) and height (by stadiometry to the nearest 0.1 cm, Seca 242, Hanover, MD) were measured in lightweight clothing. Body fat (%) and truncal fat (%) were measured by dual-energy X-ray absorptiometry (DXA; iDXA Software Encore 11.10, GE Lunar Medical Systems, Madison, WI). Fat mass (kg) and % truncal fat mass were calculated using the bodyweight and DXA measurements. Exercise performance was measured during a graded treadmill exercise test [18] with a SensorMedics Vmax Spectra 229c metabolic cart (CareFusion, San Diego, CA) for the analysis of oxygen consumption at peak exercise (VO_2_ peak). In addition, women were asked to record daily pedometer counts and track the amount of time walking and using aerobic exercise machines each day throughout the 6-month study. Activity diaries were submitted on a weekly basis.

A reduced sample insulin-modified intravenous glucose tolerance test (FSIGT) was used in conjunction with the minimal model of glucose kinetics to determine an insulin sensitivity index (S_I_) (MinMOD Millenium v6.02, Los Angeles, CA) [19, 20]. Testing was performed at 8 AM following a 12-h fast after placement of intravenous lines in both antecubital veins. After obtaining baseline glucose and insulin samples, dextrose (0.3 g/kg) was injected at time 0 over approximately1 min. A bolus of insulin (0.03 units/kg) was injected rapidly at 20 min. Blood samples were taken for determination of glucose and insulin concentrations at 0, 2, 4, 8, 19, 22, 30, 40, 50, 70, 100, and 180 min. The acute insulin response to glucose (AIRg) was determined by the area under the insulin concentration curve above basal insulin concentration between 0 and 10 min.

Leptin was measured in serum by ELISA (Millipore, Billerica, MA). The minimum detectable concentration was 0.5 ng/ml, with intraassay CV 3.7 % and interassay CV 4.0 %. Adiponectin was measured in serum by ELISA (R&D Systems, Minneapolis, MN). The minimum detectable concentration was 0.25 ng/ml, with intraassay CV 3.5 % and interassay CV 6.5 %.

**Fig. 1:**
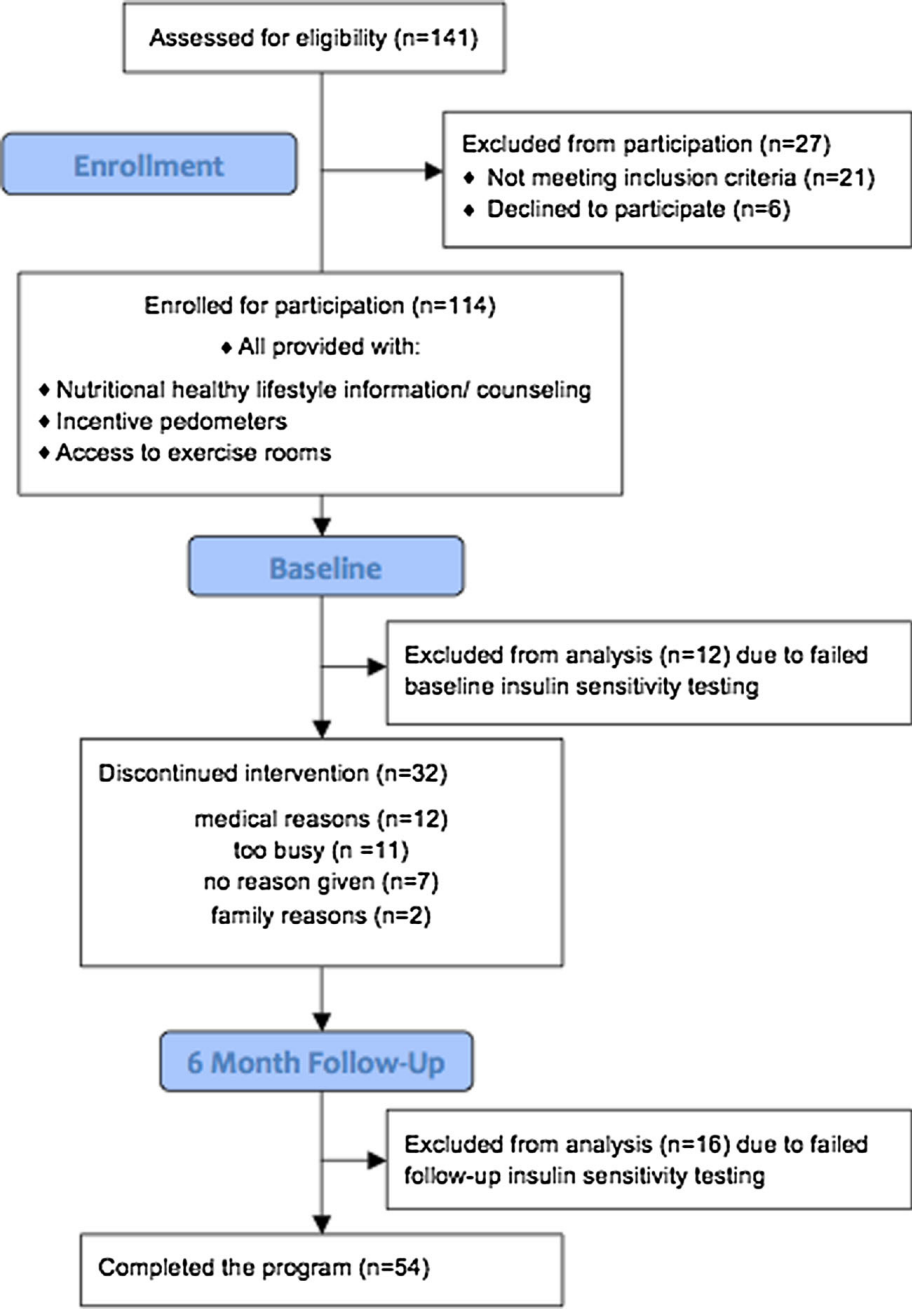
Flow chart of participant participation

One hundred forty-one black women were assessed for eligibility in this protocol, and 114 were enrolled for participation (Fig. 1). Of the 114 participants who underwent baseline testing, 28 women were excluded from analysis due to failed baseline or follow-up FSIGT. Reasons for failed FSIGT’s included hemolyzed samples (*n* = 21), inability to place intravenous lines in antecubital veins of both arms (*n* = 4), improper testing technique (*n* = 2), and poor data fit for predicted glucose disposal (*n* = 1). In addition, 32 women withdrew from the study prior to completing 6 months participation. Thus, 54 subjects underwent testing for fat mass and insulin sensitivity at baseline and at 6 months. To evaluate whether the characteristics of these 54 subjects differed from the remaining subjects who either had incomplete data or dropped out of the study (Fig. 1), we compared all baseline variables. The two groups did not differ in age or baseline BMI, total fat mass, % truncal fat mass, insulin, glucose, or S_I_ (all *P* > 0.1). Those that dropped out or with incomplete data, however, had significantly lower baseline VO_2_ peak than those who completed the study (20.7 ± 4.4 vs. 22.8 ± 4.9 mL O_2_/kg/min, *P* = 0.019).

### Statistical Analysis

As it was anticipated that not all subjects would lose weight and fat mass, it was planned that changes in insulin sensitivity would be analyzed for those who lost the most fat mass compared with those who did not lose fat mass. Accordingly, the primary analysis for this report was change in insulin sensitivity by tertile of change in fat mass for women who had measures both at baseline and at 6-month completion of the protocol by analysis of variance (ANOVA) with posthoc testing among tertiles, if statistically significant. Since no significant difference was determined in weight loss (−2.1 ± 3.5 kg vs. −1.1 ± 3.9 kg, *P* = 0.167) following program completion between those subjects randomized to attend group nutrition instructional sessions (*n* = 26) and those who were provided internet-based nutrition and exercise information only (*n* = 28), the groups were combined for this analysis. The contribution of exercise performance to insulin sensitivity at baseline and following program completion was also investigated. Paired or unpaired *t*-tests for continuous data and chi-square proportionality tests were performed using InStat biostatistics software (Version 3.06, 2003). Univariate associations among fat mass measurements, adipokines, exercise performance, and insulin sensitivity were performed using either Pearson’s correlation or Spearman’s rank correlation, as appropriate. Independent predictors of change in S_I_ following program completion were determined by multivariable regression analysis. All adjusted model analyses were performed using the SAS statistical analysis package (SAS User’s Guide: Statistics, Version 9 Edition: SAS Institute Inc, Cary, NC). Skewed data were log transformed. A *P*-value ≤0.05 was considered statistically significant. Nonskewed data are reported as mean ± standard deviation. Tertiles of fat mass change are reported as mean and range of values within each tertile.

## Results

For the 54 subjects included in the analysis for this report, the average age at baseline was 45 ± 10 years with an average BMI of 34.2 ± 6.0 kg/m^2^ (range 25.9 to 54.7 kg/m^2^); 70 % had a BMI ≥ 30 kg/m^2^. Menopausal status of the subjects was 37 premenopausal [five on hormonal contraception therapy (14 %)], 17 perimenopausal, or postmenopausal [two on hormonal therapy (12 %)]. Energy intake for the group at baseline was 2,024 ± 614 kcal/day. Baseline measures of fat mass, exercise performance, insulin sensitivity, and adipokines leptin and adiponectin are shown in Table 1.

**Table 1:** Data for 54 overweight and obese black women who completed the 6-month weight loss program

	Baseline	6 months	*P*-value
Adiposity^a^			
Weight (kg)	92.6 ± 18.1	91.1 ± 18.9	0.003
Total fat mass (kg)	40.8 ± 12.4	39.4 ± 12.6	<0.001
% Truncal fat mass (%)	46.4 ± 6.6	45.2 ± 6.7	0.003
Exercise performance^a^			
Peak VO_2_ (mL/kg/min)	22.8 ± 4.9	23.8 ± 5.3	0.028
Adipokines^a^			
Leptin (ng/ml)	61.1 ± 37.1	52.1 ± 24.2	0.027
Adiponectin (μg/ml)	5.6 ± 3.6	5.7 ± 2.9	0.389
Insulin sensitivity^a^			
Insulin (μU/ml)	7.2 ± 5.4	6.9 ± 6.4	0.077
Glucose (μU/ml)	91 ± 9	88 ± 10	0.060
Insulin sensitivity index (S_I_)	3.30 ± 1.71	3.21 ± 1.56	0.665
AIRg	529 ± 461	548 ± 455	0.335

^a^Values represented as mean ± SD

**Fig. 2:**
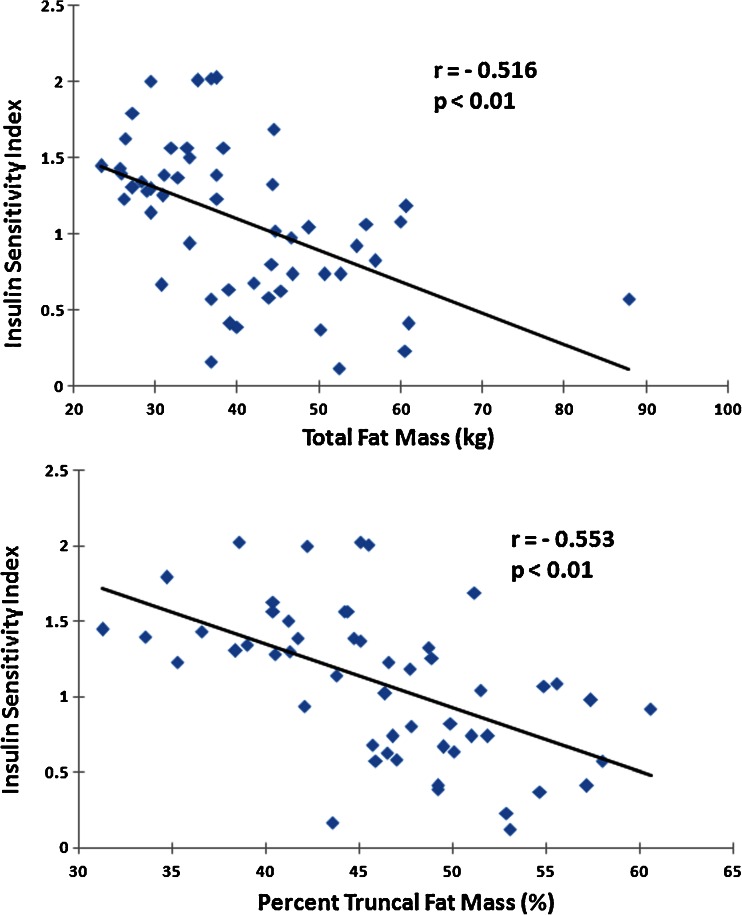
Baseline correlations between insulin sensitivity index and total fat mass (*top*) and % truncal fat mass (*bottom*), adjusted for age

At baseline, S_I_ for the 54 subjects ranged from 0.74 to 7.58 L•mU^−1^•min^−1^ and was inversely associated with total and % truncal fat masses when adjusted for age (Fig. 2). Additionally, S_I_ was positively associated with exercise performance by VO_2_ peak at baseline (*r* = 0.448, *P* < 0.001). Leptin was positively associated with total fat mass (*r* = 0.652, *P* < 0.001) and % truncal fat mass (*r* = 0.643, *P* < 0.001), and inversely associated with S_I_ (*r* = −0.492, *P* < 0.001). Adiponectin levels, however, were not related to baseline fat mass (either total or % truncal) or S_I_ (all *P* > 0.208). Endogenous insulin response, AIRg, was positively associated with total fat mass (*r* = 0.310, *P* = 0.024) but not with % truncal fat mass (*P* = 0.200).

At program completion, reduction in energy intake (−339 ± 653 kcal/day *P* < 0.001) was determined for the group, based on analyses of baseline and 6-month 3-day food records. On average, subjects reported the following activity measures: time devoted to daily walking 20 ± 24 min/day and to aerobic exercise 8 ± 8 min/day, with pedometer counts 6,131 ± 3,596 steps/day. Six-month measures of weight, fat mass, exercise performance, insulin sensitivity, and adipokine levels are shown in Table 1. Significant reductions in weight (−1.6 ± 3.7 kg, range −11.6 to +5.5 kg; *P* < 0.01), total fat mass (−1.4 ± 2.8 kg, range −10.7 to +3.8 kg; *P* < 0.01) and % truncal mass (−1.1 ± 2.7 kg, range −10.9 to +3.4 kg *P* < 0.01), improvement in VO_2_ peak (+1.0 ± 3.1 mL O_2_/kg/min, range +9.6 to −4.1 mL O_2_/kg/min; *P* < 0.001) and reduction in leptin levels (−9.1 ± 27.2 ng/mL, range −116.6 to +50.2 ng/mL; *P* < 0.001) were determined for subjects at 6 months. Improvement in S_I_ from baseline to 6-month follow-up was significantly associated with reductions in both total FM (*r* = −0.348, *P* = 0.010) and % truncal FM (*r* = −0.423, *P* = 0.001). There was no association between the change in VO_2_ peak and change in S_I_. Reduction in % truncal fat mass was associated with reduction in leptin (*r* = 0.381, *P* = 0.008). Reduction in leptin was associated with improvement in S_I_ (*r* = −0.337, *P* = 0.021). Change in AIRg showed a trend toward significance with the change in total fat mass (*r* = 0.238, *P* = 0.087) but not with the change in % truncal fat mass (*P* = 0.135) following completion of the program.

**Fig. 3:**
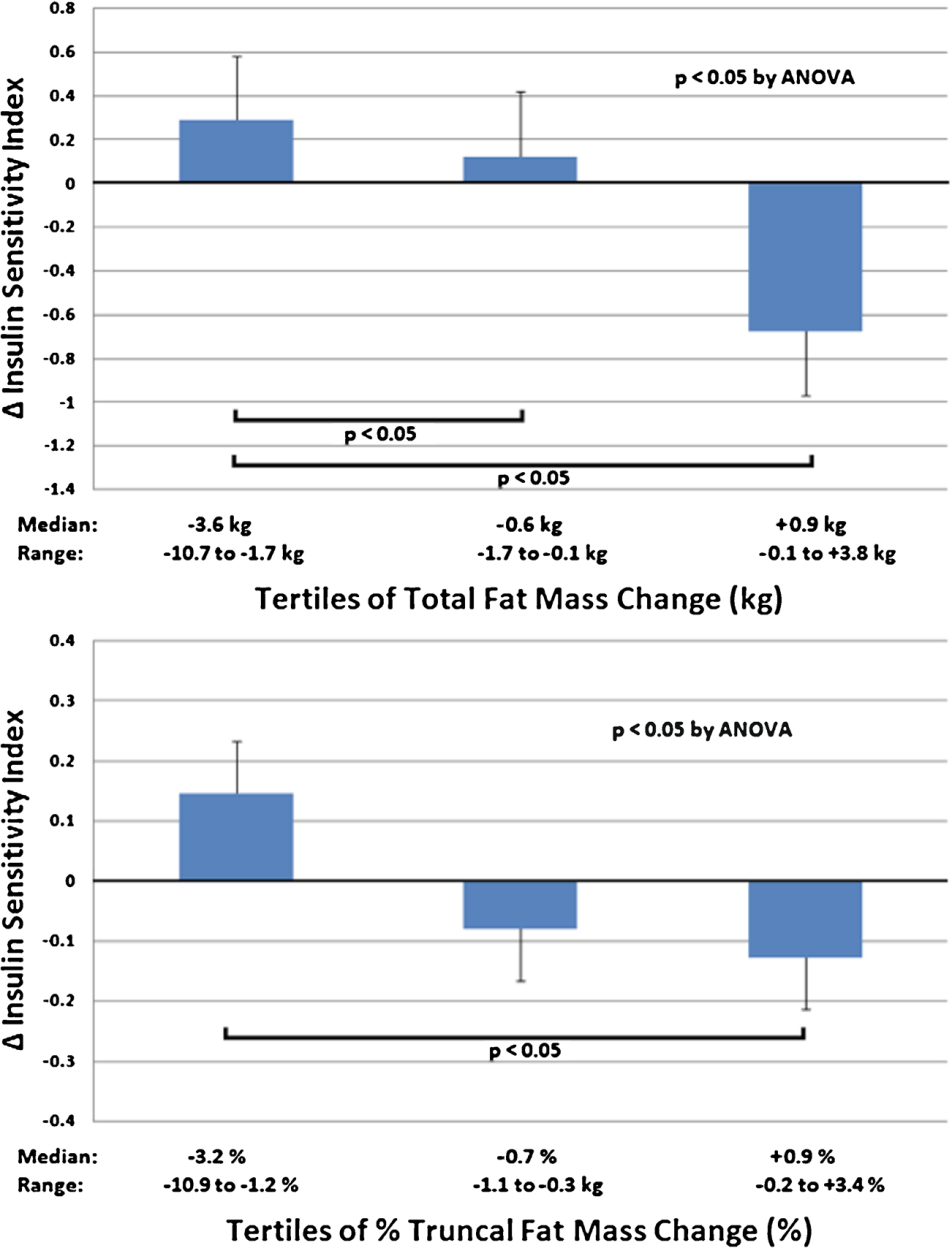
Tertiles of change in total (*upper panel*) and percent truncal fat mass (*lower panel*) and change in insulin sensitivity after completion of the 6-month weight loss program

Although on average subjects lost total fat mass and % truncal fat mass, 17 women (32 %) actually gained fat mass despite reporting energy intake reduction (−483 ± 697 vs. −287 ± 623 kcal/day), time devoted to daily walking (27 ± 25 vs. 17 ± 24 min) and aerobic exercise (7 ± 5 vs. 9 ± 10 min), and pedometer counts (5,574 ± 2,891 vs. 6,394 ± 3,895 steps/day) similar to the 37 who lost fat mass (all *P* > 0.2). Weight gain was observed with similar frequency among women who were overweight (4 of 16) or obese (11 of 38, *P* = 0.729) at the beginning of the program. There was a trend toward fewer women experiencing weight gain in the group randomized to attend nutrition instructional sessions (4 of 26) vs. those who were provided internet-based nutrition and exercise information only (11 of 28, *P* = 0.098). When participants were analyzed by tertiles of fat mass change and % truncal fat mass change, the tertile with the greatest fat mass loss improved insulin sensitivity in contrast to the tertile with net fat mass gain in which S_I_ worsened (Fig. 3). By multivariable regression analysis, changes in either total fat mass (*β* = −0.038, *P* = 0.039) or % truncal fat mass (*β* = −0.047, *P* = 0.019) were independent predictors of change in S_I_, after adjustment for age, change in leptin, and change in VO_2_ peak.

To help identify possible indicators of change in fat mass following program participation, subject characteristics of the tertile with the greatest fat mass loss were compared to the tertile with net fat mass gain. There were no significant differences between the two groups in age or baseline weight, BMI, hormonal status (premenopausal, perimenopausal, or postmenopausal), total fat mass, % truncal fat mass, VO_2_ peak, adipokine levels, glucose and insulin levels, S_I_, or AIRg (all *P* > 0.2). Although the women in the tertile with the greatest fat mass loss had a slightly greater proportion of participants in the intervention group with dietitian-led classes vs. the tertile with net fat mass gain (61 vs. 44 %), this did not achieve statistical significance (*P* = 0.317).

## Discussion

Our principal finding was that fat mass loss through participation in a worksite weight loss program can significantly improve insulin sensitivity (increase in S_I_) in overweight and obese nondiabetic black women. By multivariable regression analysis, fat mass loss – both total and truncal – was a significant predictor of increase in S_I_, independent of changes in leptin or improvement in exercise performance, both of which have been shown to improve insulin sensitivity [21–23]. We found no evidence of improved insulin secretion with fat mass loss as no difference was observed in the AIRg in the initial minutes of the FSIGT between the tertile of subjects with the greatest fat mass loss compared to the tertile with net fat mass gain. Our findings of improved insulin sensitivity with relatively modest fat mass reduction extend observations in other studies which have enrolled blacks that reported improved insulin sensitivity but with greater weight loss than achieved by most of our participants [24–26].

A disconcerting finding in our study was that nearly one-third of the overweight and obese black subjects who underwent testing for fat mass and insulin sensitivity at baseline and at 6 months actually gained fat mass with associated adverse metabolic consequences. Specifically, a decrease in insulin sensitivity was observed in the tertile of women with net fat mass gain despite self-reports of reduced caloric intake and increased physical activity similar to that of the tertile who lost the most fat mass. One reason why some of the participants gained fat mass over the course of this study despite provision of resources to enable weight loss could be loss of interest in the program. As reviewed by Fitzgibbons et al. [15], weight loss trials – including those with black women – often have high attrition similar to the nearly 30 % dropout rate observed in our study. However, the participants we report in this paper – including those who gained fat mass – all completed the 6-month program, suggesting commitment to the goals of the study. An alternative explanation for fat mass gain by many subjects in our study may be misinterpretation of the energy expenditure associated with physical activity performed by participants. Although all participants were provided with the same fitness resources including access to exercise rooms at the worksite and pedometers to incentivize exercise, those who lost fat mass had significantly greater improvement in exercise performance despite reporting similar time spent in daily aerobic activity and pedometer counts to those who gained fat mass. It is possible that those who gained fat mass either engaged in physical activity with lower energy expenditure compared with those who lost fat mass or increased caloric intake as a result of over-overestimating calories consumed with relatively low-intensity exercise. Additionally, errors in assessing – and reporting – nutritional composition of diet and daily caloric intake may have also played a role in the increased fat mass experienced by several of our participants. It is possible that the 3-day food records used at the beginning and end of each participant’s participation might not have been indicative of energy intake throughout the 6-month period. Use of more reliable food assessment tools such as the picture recording technique reported by Six et al. [27] may limit the discrepancy between the foods consumed and those reported in weight loss studies similar to ours [28].

Regardless of the reason for fat mass gain by some of our subjects, the metabolic consequence was apparent in the form of reduction in insulin sensitivity. Baseline characteristics of our participants did not identify who would lose fat mass and improve insulin sensitivity and who would gain fat mass with diminished insulin sensitivity at completion of the program. More detailed phenotyping of novel adipokines and other biomarkers involved in regulation of body weight in future work may identify mechanisms by which study participants may have been resistant to fat mass loss, as has been seen in studies of weight regain [29].

Our findings of fat mass gain with reduced insulin sensitivity in almost a third of participants indicate that some may require additional attention to achieve weight and fat mass loss. One approach that has been proposed to identify and focus on those participants that are motivated to lose weight but are not successfully doing so is a stepped-care design [30, 31]. In a stepped-care intervention, participants that are not attaining short-term weight loss goals are prescribed additional resources and nutrition counseling to help them achieve their long-term weight loss goals. Thus, Jakicic and colleagues [31] tested a stepped-care model in a randomized trial of 363 overweight and obese adults (83 % female, 33 % nonwhite) that included increased contact frequency (group sessions, mail, telephone, and individual sessions) and even meal replacements for participants missing weight loss goals at 3-month intervals. This intervention approach resulted in approximately 7 % weight loss after 18 months of participation. It is possible that fat mass loss may be achieved by the use of portable devices to self-monitor diet and physical activity as reported in the Move! Trial conducted in a Veterans Administration cohort [32]. In addition, individualized counseling or motivational interviewing might have increased compliance and achieved greater success in our participants [33].

An important message of this study is the demonstration that relatively modest fat mass loss achieved by most black women improved insulin sensitivity in a population that is at high risk of developing diabetes and associated complications. A limitation of the study is that the duration was only 6 months: Continuation for a longer period might have resulted in greater fat mass loss and further improvement in insulin sensitivity but might have been associated with even greater weight and fat mass gain with worsened metabolic consequences in some participants. In addition, as this report evaluates the change in fat mass and insulin sensitivity only in overweight and obese black women, these findings cannot be extended to men or to other racial groups.

We conclude that fat mass loss can be achieved in overweight and obese nondiabetic black women through participation in a worksite weight loss program, which can significantly improve insulin sensitivity. However, fat mass gain and worsened insulin sensitivity were also frequent among our participants despite self-reports of compliance. As a result, participation by black women in weight loss programs could paradoxically contribute to diabetes risk in some subjects, who may require special attention early in these programs to achieve success.

## References

[1] Flegal KM, Carroll MD, Ogden CL, Curtin LR (2010). Prevalence and trends in obesity among US adults, 1999-2008. JAMA.

[2] Ogden CL, Carroll MD, Kit BK, Flegal KM (2012). Prevalence of obesity in the United States, 2009-2010. NCHS Data Brief.

[3] Brancati FL, Whelton PK, Kuller LH, Klag MJ (1996). Diabetes mellitus, race, and socioeconomic status. A population-based study. Ann Epidemiol.

[4] Wilson PW, D’Agostino RB, Sullivan L, Parise H, Kannel WB (2002). Overweight and obesity as determinants of cardiovascular risk: the Framingham experience. Arch Intern Med.

[5] Brancati FL, Kao WH, Folsom AR, Watson RL, Szklo M (2000). Incident type 2 diabetes mellitus in African American and white adults: the Atherosclerosis Risk in Communities Study. JAMA.

[6] Harris MI, Flegal KM, Cowie CC, Eberhardt MS, Goldstein DE, Little RR (1998). Prevalence of diabetes, impaired fasting glucose, and impaired glucose tolerance in U.S. adults. The Third National Health and Nutrition Examination Survey, 1988-1994. Diabetes Care.

[7] Hamilton MT, Hamilton DG, Zderic TW (2007). Role of low energy expenditure and sitting in obesity, metabolic syndrome, type 2 diabetes, and cardiovasculardisease. Diabetes.

[8] McCrady SK, Levine JA (2009). Sedentariness at work: how much do we really sit?. Obesity.

[9] Hennrikus DJ, Jeffery RW (1996). Worksite intervention for weight control: a review of the literature. Am J Health Promot.

[10] Anderson LM, Quinn TA, Glanz K, Ramirez G, Kahwati LC, Johnson DB (2009). The effectiveness of worksite nutrition and physical activity interventions for controlling employee overweightand obesity: a systematic review. Am J Prev Med.

[11] Atlantis E, Chow CM, Kirby A, Fiatarone Singh MA (2006). Worksite intervention effects on physical health: a randomized controlled trial. Health Promot Int.

[12] Lemon SC, Zapka J, Li W, Estabrook B, Rosal M, Magner R (2010). Step ahead a worksite obesity prevention trial among hospital employees. Am J Prev Med.

[13] Proper KI, Koning M, van der Beek AJ, Hildebrandt VH, Bosscher RJ, van Mechelen W (2003). The effectiveness of worksite physical activity programs on physical activity, physical fitness, and health. Clin J Sport Med.

[14] Proper KI, Hildebrandt VH, Van der Beek AJ, Twisk JW, Van Mechelen W (2003). Effect of individual counseling on physical activity fitness and health: a randomized controlled trial in a workplace setting. Am J Prev Med.

[15] Fitzgibbon ML, Tussing-Humphreys LM, Porter JS, Martin IK, Odoms-Young A, Sharp LK (2012). Weight loss and African-American women: a systematic review of the behavioural weight loss intervention literature. Obes Rev.

[16] U.S. Department of Health and Human Services and U.S (2005). Department of Agriculture Dietary Guidelines for Americans, 2005 U.S.

[17] University of Minnesota. Nutrition Coordinating Center. http://www.ncc.umn.edu/products/ndsr.html.

[18] Bruce RA, Blackmon JR, Jones JW, Strait G (2004). Exercising testing in adult normal subjects and cardiac patients. 1963. Ann Noninvasive Electrocardiol.

[19] Sumner AE, Luercio MF, Frempong BA, Ricks M, Sen S, Kushner H (2009). Validity of the reduced-sample insulin modified frequently-sampled intravenous glucose tolerance test using the nonlinear regression approach. Metabolism.

[20] Boston RC, Stefanovski D, Moate PJ, Sumner AE, Watanabe RM, Bergman RN (2003). MINMOD Millennium: a computer program to calculate glucose effectiveness and insulin sensitivity from thefrequently sampled intravenous glucose tolerance test. Diabetes Technol Ther.

[21] Holloszy JO (2005). Exercise-induced increase in muscle insulin sensitivity. J Appl Physiol.

[22] Considine RV, Premkumar A, Reynolds JC, Sebring NG, Ricks M, Sumner AE (2008). Adiponectin and leptin in African Americans. Obesity.

[23] Rabe K, Lehrke M, Parhofer KG, Broedl UC (2008). Adipokines and insulin resistance. Mol Med.

[24] Gower BA, Weinsier RL, Jordan JM, Hunter GR, Desmond R (2002). Effects of weight loss on changes in insulin sensitivity and lipid concentrations in premenopausal African American and white women. Am J Clin Nutr.

[25] Goodpaster BH, Kelley DE, Wing RR, Meier A, Thaete FL (1999). Effects of weight loss on regional fat distribution and insulin sensitivity in obesity. Diabetes.

[26] Racette SB, Weiss EP, Obert KA, Kohrt WM, Holloszy JO (2001). Modest lifestyle intervention and glucose tolerance in obese African Americans. Obes Res.

[27] Six BL, Schap TE, Zhu FM, Mariappan A, Bosch M, Delp EJ (2010). Evidence-based development of a mobile telephone food record. J Am Diet Assoc.

[28] Schoeller DA, Thomas D, Archer E, Heymsfield SB, Blair SN, Goran MI (2013). Self-report-based estimates of energy intake offer an inadequate basis for scientific conclusions. Am J Clin Nutr.

[29] Sumithran P, Prendergast LA, Delbridge E, Purcell K, Shulkes A, Kriketos A (2011). Long-term persistence of hormonal adaptations to weight loss. N Engl J Med.

[30] Brownell KD (1986). Public health approaches to obesity and its management. Annu Rev Public Health.

[31] Jakicic JM, Tate DF, Lang W, Davis KK, Polzien K, Rickman AD (2012). Effect of a stepped-care intervention approach on weight loss in adults: a randomized clinical trial. JAMA.

[32] Spring B, Duncan JM, Janke EA, Kozak AT, McFadden HG, DeMott A (2013). Integrating technology into standard weight loss treatment: a randomized controlled trial. JAMA Intern Med.

[33] Armstrong MJ, Mottershead TA, Ronksley PE, Sigal RJ, Campbell TS, Hemmelgarn BR (2011). Motivational interviewing to improve weight loss in overweight and/or obese patients: a systematic review and meta-analysis of randomized controlled trials. Obes Rev.

